# Post-cesarean scar endometriosis

**DOI:** 10.4274/tjod.90922

**Published:** 2018-03-29

**Authors:** Doğan Yıldırım, Cihad Tatar, Ozan Doğan, Adnan Hut, Turgut Dönmez, Muzaffer Akıncı, Mehmet Toptaş, Rahime Nida Bayık

**Affiliations:** 1 University of Health Sciences, Haseki Training and Research Hospital, Clinic of General Surgery, İstanbul, Turkey; 2 Şişli Hamidiye Etfal Training and Research Hospital, Clinic of Obstetrics and Gynecology, İstanbul, Turkey; 3 Lutfiye Nuri Burat State Hospital, Clinic of General Surgery, İstanbul, Turkey; 4 University of Health Sciences, Haseki Training and Research Hospital, Clinic of Anestehesiology, İstanbul, Turkey; 5 Bahçeşehir University Faculty of Medicine, Göztepe Medical Park Hospital, Department of Obstetrics and Gynecology, İstanbul, Turkey

**Keywords:** Cesarean, endometriosis, scar

## Abstract

**Objective:**

Endometriosis is seen in women during their reproductive period, where stromal tissue and functional endometrial glands of the uterus are observed outside the uterine cavity. In this study, we aimed to identify the clinical characteristics of our patients who underwent surgery with scar endometriosis and to discuss the surgical results in light of the literature.

**Materials and Methods:**

A total of 24 patients who underwent surgery and diagnosed as having endometriosis as the result of a pathologic examination were retrospectively evaluated.

**Results:**

The mean age of the patients was 31 years. Thirteen presented to general surgery and 11 presented to gynecology outpatient clinics. The pain was cyclical in 19 patients. There was history of cesarean section in 9 patients, twice in 12, and 3 times in three patients. The mean diameter was 39.1 mm on ultrasound, and 37.5 mm on magnetic resonance imaging. Endometriosis was on the left side of the incisions in 13, whereas it was on the right in 11. The mean weight of the lesions was 61.6 grams.

**Conclusion:**

The occurrence of endometriosis is supported by the iatrogenic implantation theory. In the event of a mass in the abdominal wall, previous obstetric and gynecologic operations and a history of a painful mass during menstruation periods must be questioned. In the treatment of scar endometriosis, excision is required by obtaining secure margins. If diagnosis can be established preoperatively, unnecessary surgeries can prevented.

**PRECIS:** Scar endometriosis is a condition seen in women during their reproductive period, excision is required by obtaining secure margins in the treatment.

## Introduction

Endometriosis is a condition seen in women during their reproductive period in which both the stromal tissue and the functional endometrial glands are observed outside the uterine cavity. It mostly occurs through iatrogenic seeding in the wake of obstetric and gynecologic surgeries. Patients generally present to general surgery clinics^(1)^. Although endometriosis is often found in the pelvic cavity, it may also show localization outside the pelvic region, such as the heart, lungs, liver, kidneys, central nervous system, and the abdominal wall. Even though the endometrium is found in areas outside its normal localization, endometriotic foci still contain normal endometrial tissue. For this reason, they perform a menstrual cycle as an organ functioning within itself. During menstrual periods, thickening, destruction, and menstrual bleeding also occur almost always in these areas, just as in the endometrium. Perhaps the only difference of scar endometriosis from the endometrial tissue inlaid within the uterus is that it fails to drain the blood formed there. 

Though several theories have been reported as to its formation, the theory of direct implantation is the most recognized^(2)^. Ectopic endometriosis foci do not generally show the tendency to become malignant^(3)^. Various broad series in the literature have addressed why the incidence of the disease in question is rare^(4)^. In this study, we aimed to identify the clinical characteristics of our patients who underwent surgery due to scar endometriosis and to discuss the surgical results in light of the literature. 

## Materials and Methods

Data of 29 patients who underwent surgery in the Clinic of General Surgery Haseki Training and Research Hospital between January 2012 and June 2016 with preoperative diagnoses of scar endometriosis, which were confirmed by pathology, were retrospectively examined. 

Of the 29 patients, five were excluded from the study due to a lack of abdominal magnetic resonance imaging (MRI). All the demographic data, symptoms at the time of presentation, imaging reports including both ultrasonography (USG) and MRI, and pathology reports were retrospectively reviewed. The study was approved by the Haseki Training and Research Hospital Local Ethics Committee (approval number: 367). Informed consent was obtained from all subjects.

### Statistical Analysis

Data concerning demographic and clinical characteristics were analyzed using descriptive methods (means, minimum-maximum). The statistical software used was SPSS for Windows, version 15.0 (SPSS Inc., Chicago, IL, USA).

## Results

The mean age of the patients was 31 years (range, 21-40 years). Thirteen (54.2%) patients had presented to the general surgery outpatient clinic and 11 (45.8%) had presented to the gynecology clinic. Twenty-one (87.5%) patients had a painful mass in their previous surgery area, and three (12.5%) had pain only in their previous surgery area. The pain was cyclical in 19 (79.2%) of the patients, whereas it was non-cyclical in six (20.8%). The mean duration of symptoms was 19.8 months (range, 9-31 months). The number of previous ceserean sections in the subjects were as follows: one section in nine patients (37.5%), two in 12 patients (50%), and three in three patients (12.5%). The mean greatest diameter of the endometriotic masses was 39.1 mm (range, 21-54 mm) on USG, and 37.5 mm (range, 21-55 mm) on MRI. 

**Figure 1:**
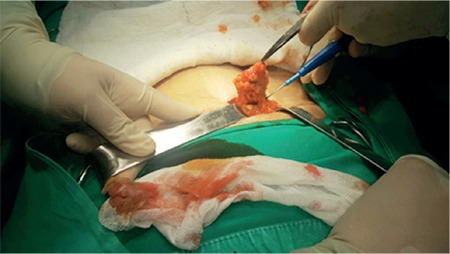
Excision of scar endometriosis

Endometriosis was detected on the left side of the incisions of 13 patients (54.2%), whereas it was found on the right side of the incisions of 11 (45.8%) patients. A solid heterogeneous mass detected in 22 (91.6%) patients, an incisional hernia was detected in one (4.2%), and a mass sporadically containing a solid area suggestive of an abscess was detected in one (4.2%). Three (12.5%)  patients underwent surgery due to incisional hernia, whereas 21 (87.5%) patients underwent surgery due to the pre-diagnoses of a tumour on the anterior abdominal wall (Figure 1). During the operations of patients with pre-diagnoses of incisional hernia, a hernia sac and masses adhered to the hernia sac were detected. The masses along with the hernia sac were excised. The defects formed as the result of excising the mass were sutured primarily and then repaired. As the result of the pathology examination, it was reported that all lesions were accordant with endometriosis and that the mean weight of the excised lesions was 61.6 g (range, 46-73 g). None of the patients had any post-operative complications. The patients’ mean hospitalizaiton period was 2 days (range, 1-5 days). The mean follow-up period was 22 months (range, 6-51 months), and no recurrence was detected in any patients (Table 1).

## Discussion

Endometrioma is defined as endometriosis that forms a mass with a smooth boundary. Although scar endometriosisis can be seen after cesarean surgeries, it may also develop after hysterectomy, hysterotomy, tubal surgeries, appendectomy, trocar-site, amniocentesis, and episiotomy^(5,6,7)^. There have been many theories put forward in terms of its etiopathogenesis. Scar endometriosis is accepted to be formed through the iatrogenic auto-transplantation of endometrial cells during surgery^(8,9,10)^. It can be seen in the lungs, liver, kidneys, ureters, central nervous system, abdominal scar tissues, and in the extremities, apart from in pelvic organs^(2,4)^. Even though scar endometriosis may occur months^(11)^ and even years after gynecologic surgery, the mean occurrence period is 30 months. 

Elabsi et al.^(12)^ reported an abdominal wall endometrioma that occurred in the wake of a cesarean surgery performed 22 years previously. In our series, the postsurgical period of the patients was 19.8 months (range, 9-31 months) on average. 

The incidence of scar endometriosis has been reported to be between 0.03% and 1.7%^(13)^. The most frequent finding is cyclical or non-cyclical painful mass^(14,15)^. At the onset of symptoms, patients are often diagnosed as having inguinal hernia, incisional hernia, and abdominal wall tumors, after which they may be exposed to unnecessary interventions. Accordingly, failure to perform the necessary treatment, or any delay in performing the treatment may also cause emotional and physical stress in patients^(16)^. During the definitive diagnosis; lipoma, granuloma, sebaceous cyst, neuroma, hernia, hematoma, lymphadenopathy, lymphoma, desmoid tumors and sarcomas on the abdominal wall must also be considered^(2,4,17)^. In our study, 13 (54.2%) patients presented to general surgery clinics and 11 (45.8%) presented to gynecology outpatient clinics. 

Andolf et al.^(18)^ in their prospective study in which 578.785 patients were incorporated, detected endometriosis in 749 of 130.305 (0.6%) patients who had given birth through cesarean section. In their study, it was reported that there was no difference in terms of the risk of the development of endometriosis between those who underwent cesarean once and those who had a history of more than one cesarean; however, it was also reported that the risk of endometriosis in those who gave birth through cesarean section was twice as much when compared with those who had vaginal birth. In our series, there was a history of cesarean section in all patients, none had had vaginal births. 

**Figure 2:**
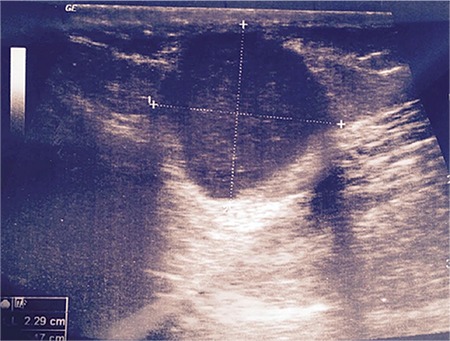
Ultrasonography

**Figure 3:**
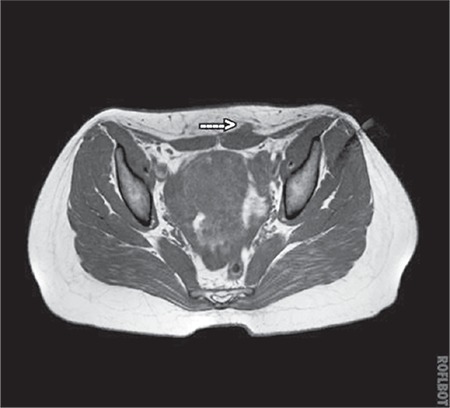
Magnetic resonance image axial

**Figure 4:**
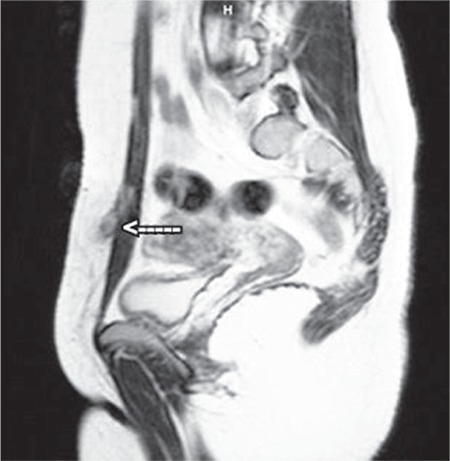
Magnetic resonance image coronar

In the evaluation of the mass, USG, computed tomography, and MRI are not examinations that establish the final diagnosis, but provide information about the location of the mass as well as its size and volume. USG and MRI are often the preferable methods for diagnosis (Figure 2,3,4). Differences in size in radiologic imaging may vary depending on the day of the menstrual cycle, the ratio of stromal and glandular elements, the amount of bleeding, and the inflammatory response in the peripheral tissue. Though hypoechoic, a vascularized nodule is seen in USG, it can also be seen as cystic, polycystic or heterogeneous echo. The advantage of MRI over USG is its ability to distinguish masses that imitate endometriosis on the abdominal wall. 

**Figure 5:**
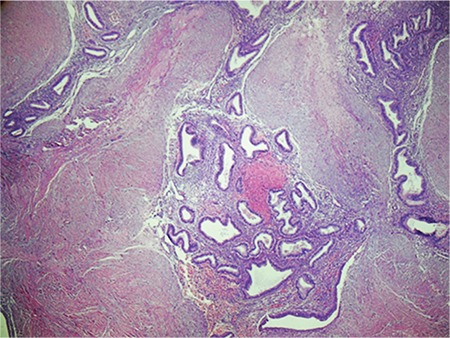
The pathologic detection of glandular epithelial cells, oval stromal cells

In general, biopsy is not required because anamnesis, physical examination, and imaging methods are adequate in the diagnosis of endometriosis. Biopsy is only performed under conditions in which malignancy is suspected. The diagnosis-establishing value of fine needle aspiration biopsy is low. Nevertheless, it is still recommended in scar endometriosis due to its convenience. For a final diagnosis, an accurate pathologic analysis is required. The pathologic detection of glandular epithelial cells, spindle or oval stromal cells, and hemosiderin-laden macrophages allows for establishing a diagnosis^(19,20)^ (Figure 5). On the other hand, because incisional biopsy will cause endometriosis to spread even further, some studies have advised against performing this procedure^(2,3,4,21)^. In our study, neither incisional biopsy nor fine needle aspiration was performed. 

Apart from the fact that there are two types of treatment options in pelvic cases, which involve medical and surgical methods, neither of them is an effective method of treatment on its own. However, surgery in scar endometriosis, as is also seen in our surgical series, is the gold standard treatment approach. Medical treatment, on the other hand, must be reserved for patients who cannot undergo surgery. 

Surgical excision with at least a 1 cm margin boundary should be performed to prevent recurrence in surgical treatment, in addition, some part of the neighbouring structures, such as fascia or muscle, also needs to be excisedl. Thus, the recurrence of endometriosis in the wound area will be prevented by means of the transplantation of microscopic endometrial tissue residuals. In the event that the invasion depth into structures of the abdominal wall causes large defects after surgery, a repair with synthetic materials should be performed^(22,23)^. In our case series, a large excision was performed in 5 patients because the masses had invaded the peritoneal surface, and the large defect area formed on the abdominal wall after the operation was supported with Prolene mesh. If no residual tissue is left inside in scar endometriosis, no additional treatment is required. The recurrence rates after total excision is quite low. In our series, no recurrence was seen during follow-up. 

### Study Limitations

The small patient population and the retrospective nature of the study are our limitations.

## Conclusion

The occurrence of endometriosis whose etiopathogenesis has not yet been fully explained is supported by most authors through the theory of iatrogenic implantation. In the patients who visit hospital with symptoms of a mass on the abdominal wall, previous obstetric and gynecologic operations as well as the medical history of a painful mass becoming increasingly severe during menstruation periods must be questioned in full. In the treatment of scar endometriosis, excision is required by obtaining a secure marginal boundary. If diagnosis can be established in advance in scar endometriosis, then the performance of unnecessary surgeries will be prevented. Studies in broad series are needed to be conducted on such diseases that are rarely observed.

## Figures and Tables

**Table 1:**
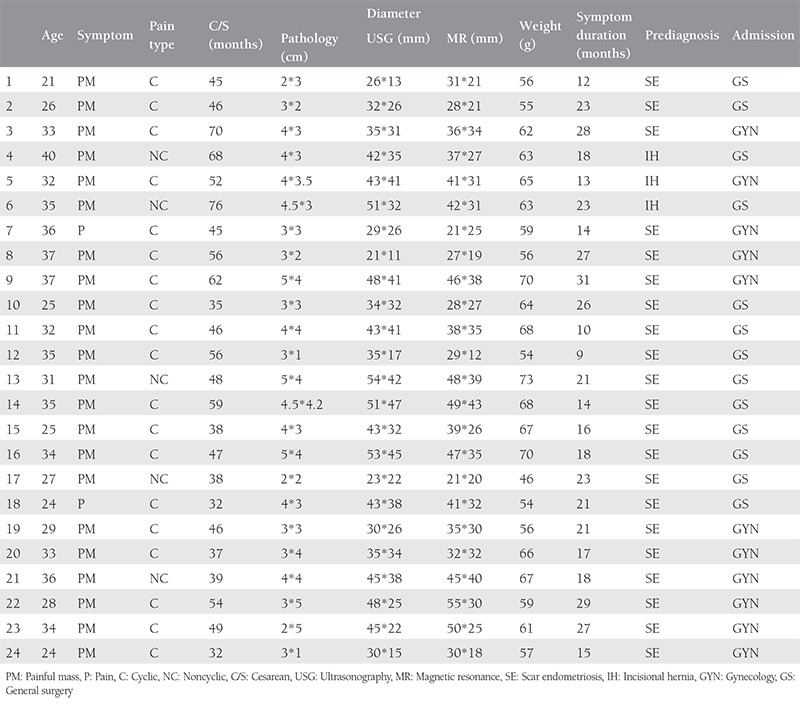
Patient’s demographics
